# Age- and sex-related differences of muscle cross-sectional area in iliocapsularis: a cross-sectional study

**DOI:** 10.1186/s12877-022-03127-y

**Published:** 2022-05-18

**Authors:** Masahide Yagi, Masashi Taniguchi, Hiroshige Tateuchi, Tetsuya Hirono, Yoshihiro Fukumoto, Momoko Yamagata, Ryusuke Nakai, Yosuke Yamada, Misaka Kimura, Noriaki Ichihashi

**Affiliations:** 1 Human Health Sciences, Graduate School of Medicine, Kyoto University, 53 Kawahara-cho, Shogoin, Sakyo-ku, Kyoto, 606-8507 Japan; 2 Research Fellow of Japan Society for the Promotion of Science, Kojimachi Business Center Building, 5-3-1 Kojimachi, Chiyoda-ku, Tokyo, 102-0083 Japan; 3 School of Health and Sport Science, Chukyo University, 101 Tokodachi, Kaizu-cho, Toyota, Aichi 470-0393 Japan; 4 Faculty of Rehabilitation, Kansai Medical University, 18-89 Uyama Higashimachi, Hirakata, Osaka 573-1136 Japan; 5 Institute for the Future of Human Society, Kyoto University, 46 Shimoadachi-cho, Yoshida Sakyo-ku, Kyoto, 606-8501 Japan; 6 National Institutes of Biomedical Innovation, Health and Nutrition, 1-23-1, Toyama, Shinjuku-ku, Tokyo, 162-8636 Japan; 7 Institute for Active Health, Kyoto University of Advanced Science, Nanjo Otani, 1-1 Sogabecho, Kameoka, Kyoto, 621-8555 Japan; 8 Faculty of Nursing, Doshisha Women’s College of Liberal Arts, Koudo, Kyotanabe, Kyoto, 610-0395 Japan

**Keywords:** Age-related difference, Atrophy, Iliocapsularis, Sex-related difference

## Abstract

**Background:**

This study aimed to determine in how many individuals the iliocapsularis muscle (IC) could be identified on magnetic resonance imaging (MRI) and whether age and sex are associated with the cross-sectional area (CSA) of the IC.

**Methods:**

Thirty-seven healthy younger adults and 40 healthy older adults were assigned to four groups: 1) 20 younger men; 2) 17 younger women; 3) 20 older men; and 4) 20 older women. The CSAs of the IC, IP, the rectus femoris (RF) and the quadriceps (QUAD) were quantified on an axial MRI.

**Results:**

The number of individuals with the identified IC was *n* = 17 (85.0%) of 20 younger men, *n* = 15 (88.2%) of 17 younger women, *n* = 18 (90.0%) of 20 older men, and 19 (95.0%) of 20 older women. Our results showed the main effect of sex, but not age, in the CSA of the IC. The men-groups had larger CSA of the IC than the women-groups; however, no difference in CSA of the IC was found between the younger and older groups. Meanwhile, the main effects of age and sex were found for the IP, RF, and QUAD; thus, younger or men groups have larger CSAs of the three muscles than the older or women groups. The IC muscle can be discriminated in 85% – 95% of healthy individuals.

**Conclusion:**

Although sex and age are associated with the CSA of lower-limb muscles other than the IC, only sex is associated with the CSA of the IC.

## Background

Hip instability could potentially cause hip joint diseases, and, thus, recent studies have focused on which factors could be related to hip stability [1]. Hip stability is coordinately maintained by static stability with joint structures and dynamic stability with muscle function [2, 3]. Many studies have been conducted on static stability and have revealed essential joint structures and appropriate treatments [3–5]. However, only a few studies have investigated dynamic stability [6, 7]; hence, one of the topics was to explore the muscle function involved in dynamic stability.

The iliocapsularis muscle (IC) attaches to the anterior hip capsule and can contribute to dynamic hip stability [6, 8]. For example, IC size measured using magnetic resonance arthrography (MRA) was larger in patients with hip dysplasia, who had low osseous stability, than in healthy individuals [6, 9, 10]. The dynamic stability induced by IC is assumed to compensate for the poor static stability in these patients.

MRI and anatomical studies have reported that IC could be identified and measured in all individuals [6, 8]. Although connective tissue formed a border between the IC and the iliopsoas muscle (IP) [8], some studies have reported that the border is unclear, and these two muscles are often depicted as one muscle group on magnetic resonance imaging (MRI) [11–13]. When evaluating the hip joint in clinical situations, the percentage of individuals with an IC that was observable on MRI is little known [13].

The IC size is often used as a biomarker for hip stability [6, 10]; however, the factors affecting its size are not fully understood. Although most muscles are larger in men than in women, age-related changes also affect the size of each muscle. Indeed, the extent to which a muscle atrophies with age depends upon the fiber composition and function [14–17]. For example, age-related differences are less likely to occur in the soleus muscle, which has a high percentage of type I fibers [14], and in the transverse abdominis muscle, which functions as a trunk stabilizer to maintain body position [15]. Since the IC is a deep muscle involved in joint stability, similar to the soleus and transverse abdominis muscles, the IC size may also not decrease with age. In a previous study that reported a large IC in patients with hip disease, the experimental group differed from the control group regarding different characteristics, including age and sex [10]. To date, data available on age- and sex-related differences in the IC size are scanty [13]. These well-unknown differences may lead to misunderstanding regarding the patient-specific size of the IC.

Therefore, we posed the following questions for our research: (1) In how many individuals could the IC be identified on MRI? (2) Are age and sex associated with cross-sectional area (CSA) of the lower limb muscles, such as IC, IP, rectus femoris (RF), and quadriceps (QUAD)? We hypothesized that only the CSA of the IC was associated with sex, but not with age; that is, the IC was larger in men than in women, while the CSA of the other three muscles was associated with age and sex and was larger in younger adults and men.

## Methods

This study was a cross-sectional observational design.

### Subjects

Ninety-six community-dwelling older adults and 43 healthy university students were recruited over 3 months to investigate age-related muscle degeneration. For this study, the inclusion criteria were: absence of pain in the right leg during gait, and the ability to live independently. Exclusion criteria included appreciable deformation of the hip joint on MRI, severe musculoskeletal or neurological disease, and a degree of cognitive decline in which the individual is unable to understand the procedure explained in the informed consent.

According to the abovementioned criteria, 20 younger men, 17 younger women, 20 older men, and 20 older women were selected, and were assigned to one of four groups: Younger-Men, Younger-Women, Older-Men, Older-Women. Individuals in the older men and women groups were aged > 60 years, and those in the younger men and women groups were aged < 40 years [18, 19], while individuals aged 40–60 years were not recruited. In general, the effects of age and sex on muscle size are large, and many studies show that the size of many muscles is larger in men or younger individuals [20, 21]. Thus, we set the large effect size to calculate a required sample size for two-way analysis of variance (ANOVA), and the number was estimated from an α error of 0.05, power of 0.80, and effect size of 0.4 using G*power (Heinrich Heine University, Dusseldorf, Germany). As a result, the overall sample size was 52 individuals (13 individuals in each group), and, thus, the number of selected individuals (77 in total) met the required sample size. Table 1 summarizes the demographic data of the individuals in each group.

**Table 1:** Overview of the study events for each participant during the follow-up period

	Younger-Men (*n* = 20)	Younger-Women (*n* = 17)	Older-Men (*n* = 20)	Older-Women (*n* = 20)
Age (years)	27 (20–39)	27 (20–38)	76 (60–87)	73.5(62–84)
Height (cm)	170(160–178)	160(155–169)	165.4(153.4–176.6)	152.7(141–165)
Weight (kg)	63.5(52–73)	53(43–65)	59.6(48.9–80.9)	47.6(40–60.8)

Data are medians, with minimum-maximal values in parentheses.

Before initiating the study, the inherent procedures and goals were verbally explained, and all individuals provided written informed consent. The Ethics Committee of the Kyoto University Graduate School and Faculty of Medicine approved this study (Protocol Identification Number: R1746). The study was conducted in accordance with the Declaration of Helsinki.

### Magnetic Resonance Imaging

The T-1 weighted images of the right leg were obtained using a 3.0 T MRI scanner (MAGNETOM Verio; Siemens AG, Germany) with a body matrix coil and a spine coil. This multi-slice sequence with a slice thickness of 4 mm was performed with the following parameters: a repetition time of 2820 ms, echo time of 16 ms, field of view of 320 × 240 mm, flip angle of 129°, and voxel size of 0.5 × 0.5 × 4.0 mm. After more than 15 min of rest in a supine position, axial MRI was acquired from the pelvis to the right thigh in two measurements. The individuals were in the supine position, with their legs fixed with a wooden device and their right lower joints in the neutral position (defined with a goniometer). A reflective marker was provided to the midpoint between the anterior superior iliac spine and the superior edge of the patella.

**Fig.1:**
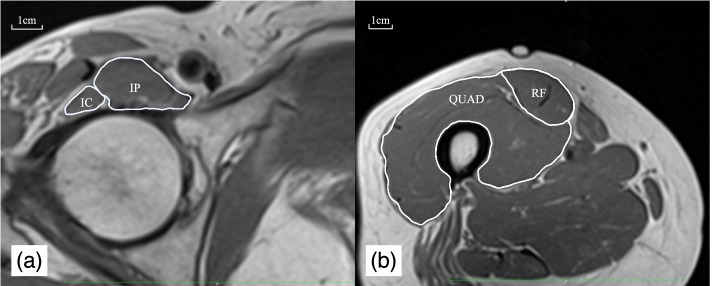
Example muscle cross-sectional area images from a reprehensive individual. IC; Iliocapsularis, IP; Iliopsoas, RF; rectus femoris, QUAD; quadriceps

The CSA was measured by an experienced examiner using Osirix MD (version 11.0; OsiriX, Geneva, Switzerland), which was based on the area surrounding the fascia. First, we identified the center of the femoral head by drawing the best-fit circle on it in the coronal plane [13, 22]. Then, following the method used in previous studies [10, 23], the CSA of the IC and the IP were quantified on axial MRI at the center of the femoral head (Fig. 1a). The CSA of RF and QUAD were quantified at the marker point as mentioned above (Fig. 1b). The CSA of the IP, the RF, and the QUAD were also measured to compare the age- and sex-related characteristics of the IC with those of other muscles in the lower extremity. We selected these muscles because the IP is close to the IC, the RF is a hip flexor like the IC, and the QUAD is often used as a representative of lower extremity muscles. Normalized CSA was calculated by dividing the CSA of each muscle by body weight in each individual [16].

Fifteen individuals were randomly selected from individuals with all four muscles identified on MRI, and the same examiners re-measured the CSA of each muscle. A one-way random effects model was used for the intraclass correlation coefficients (ICC1,1), which were 0.968 for the IC, 0.995 for the IP, 0.993 for the RF, and 0.999 for the QUAD, indicating high intra-examiner reliability for CSA measurements.

### Statistical analysis

After examining whether IC could be confirmed on MRI, the identification rate was compared among the groups using Fisher's exact test. This analysis was conducted using R version 3.6.3 for Mac OS.

The normality of CSA and normalized CSA of 4 muscles in each group was checked using the Shapiro–Wilk test. Two-way analysis of variances (ANOVAs) (sex × age) was performed for CSA and normalized CSA of each muscle. In addition, partial *η*^2^ was calculated as the effect size for ANOVA. We also performed linear regressions to determine the relationship between body weight and CSA of each muscle, and conducted the Mann–Whitney U test to compare differences in body weight between the younger and older groups and women’s and men’s groups.

Additionally, linear regressions of age and CSA and normalized CSA of each muscle were conducted for each sex and all individuals. All statistical analyses were performed using IBM SPSS Statistics 22 (IBM SPSS). The level of significance was set at *p* < 0.05.

## Results

### In how many individuals could the IC be identified using MRI?

**Fig. 2:**
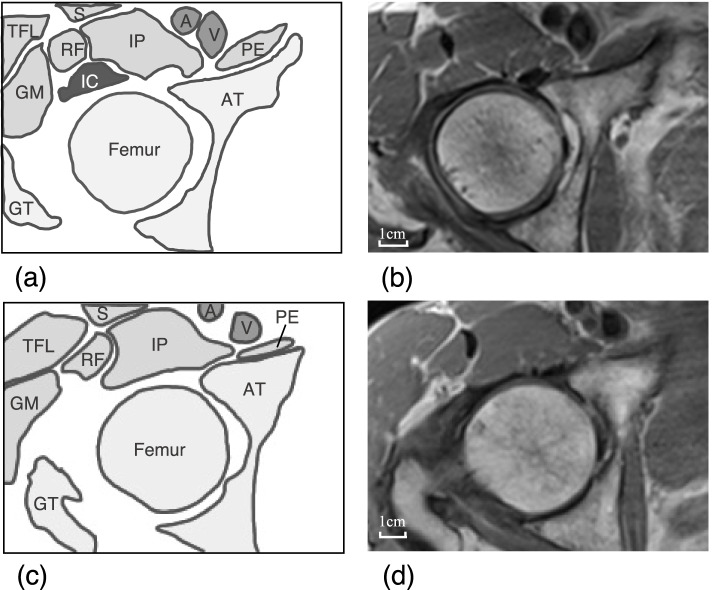
Examples of MRI images from two representable individuals. a and b were imaged from an individual who can be observed IC. c and d were imaged from another individual who cannot be observed IC. This figure was developed by referring to a previous study [6]. GM; Gluteus minimus, IC; Iliocapsularis, IP; Iliopsoas, PE; Pectineus, RF; Rectus femoris, S; Sartorius, TFL; tensor fasciae latae, Femur; femoral head, GT; greater trochanter, AT; acetabular, A; femoral artery, V; femoral vein

Examples of two representative individuals are shown in Fig. 2. The IC was identified in one individual on MRI (Fig. 2a, b), while it was not in another (Fig. 2c, d). The number of individuals who could not be identified on MRI was 3 in the Younger-Men group, but 2 in each remaining group. The identification rate was 85.0% (*n* = 17/20) in the Younger-Men group, 88.2% (*n* = 15/17) in the Younger-Women group, 90.0% (*n* = 18/20) in the Older-Men group, and 95.0% (*n* = 19/20) in the Older-Women group. Fisher's exact test showed no group-difference in these rates (*p* = 0.86).

For CSA comparison, the data of individuals in which the IC was identified were analyzed; thus, there were 17, 15, 18, and 19 individuals in the Younger-Men, Younger-Women, Older-Men, and Older-Women groups, respectively. Table 2 shows the values of each muscle CSA. The Shapiro–Wilk test indicated all data normality, except for CSA and normalized CSA of QUAD.

**Table 2:** Results of muscle cross-sectional area

		Younger-Men (*n* = 17)	Younger-Women (*n* = 15)	Older-Men (*n* = 18)	Older-Women (*n* = 19)
IC	CSA	1.6 ± 0.4	1.1 ± 0.2	1.4 ± 0.3	1.1 ± 0.3
	Normalized CSA	0.025 ± 0.006	0.020 ± 0.004	0.023 ± 0.005	0.023 ± 0.005
IP	CSA	8.1 ± 1.1	5.4 ± 1.1	5.8 ± 1.2	3.9 ± 0.7
	Normalized CSA	0.131 ± 0.019	0.102 ± 0.023	0.094 ± 0.014	0.083 ± 0.012
RF	CSA	12.0 ± 2.0	8.5 ± 2.2	8.7 ± 1.8	6.1 ± 1.1
	Normalized CSA	0.193 ± 0.030	0.159 ± 0.034	0.143 ± 0.031	0.128 ± 0.024
QUAD	CSA	69.9 ± 7.6	48.9 ± 7.6	57.4 ± 9.1	39.3 ± 6.0
	Normalized CSA	1.13 ± 0.083	0.918 ± 0.098	0.943 ± 0.151	0.829 ± 0.121

CSA (cm^2^) and normalized CSA (cm^2^/kg) were shown by mean value ± standard deviation

*IC* Iliocapsularis, *IP* Iliopsoas, *RF* Rectus femoris, *QUAD* quadriceps, *CSA* cross-sectional area

### Are age and sex associated with the CSA of the lower limb muscles?

As shown in Table 3, two-way ANOVA for CSA of the IC indicated a significant main effect for sex (*p* < 0.001; partial *η*^2^ = 0.31), but not for age (*p* = 0.27; partial *η*^2^ = 0.02). Specifically, CSA of the IC in men groups was larger than that in women groups; however, no difference was found between the younger and older groups. On the other hand, the main effects of age and sex were found for the IP (sex: *p* < 0.001, partial *η*^2^ = 0.55; age: *p* < 0.001, partial *η*^2^ = 0.46), RF (sex: *p* < 0.001, partial η^2^ = 0.41; age: *p* < 0.001, partial η^2^ = 0.39), and QUAD (sex: p < 0.001, partial η^2^ = 0.63; age: *p* < 0.001, partial *η*^2^ = 0.36); thus, CSA in these three muscles was larger in younger or men groups than in the older or women groups. Additionally, linear regression analyses showed that CSA of all muscles excluding the IC was significantly associated with age, but no association was found between CSA of the IC and age. The results were consistent between the analysis of all individuals and the analysis of each sex (Table 4).

**Table 3:** Doses escalation during the study follow up

	Cross-sectional area						Normalized cross-sectional area					
	*age*			*sex*			*age*			*sex*		
	*F*	*p*	*partial η*^*2*^	*F*	*p*	*partial η*^*2*^	*F*	*p*	*partial η*^*2*^	*F*	*p*	*partial η*^*2*^
IC	1.3	0.27	0.02	**29.8**	** < 0.001**	**0.31**	0.003	0.96	<0.01	**5.1**	**0.03**	**0.07**
IP	**56.9**	** < 0.001**	**0.46**	**81.9**	** < 0.001**	**0.55**	**42.7**	** < 0.001**	**0.39**	**20.7**	** < 0.001**	**0.24**
RF	**42.5**	** < 0.001**	**0.39**	**46.6**	** < 0.001**	**0.41**	**32.1**	** < 0.001**	**0.33**	**10.3**	**0.002**	**0.14**
QUAD	**36.3**	** < 0.001**	**0.36**	**112.2**	** < 0.001**	**0.63**	**23.2**	** < 0.001**	**0.26**	**30.7**	** < 0.001**	**0.32**

Bold font indicates significance

*IC* Iliocapsularis, *IP* Iliopsoas, *RF* Rectus femoris, *QUAD* quadriceps

**Table 4:** Results of linear regression with age

	group	Cross-sectional area				Normalized cross-sectional area			
		*B*	*95%CI*	*β*	*p*	*B*	*95%CI*	*β*	*p*
IC	All	-2.0 × 10^–3^	-6.0 × 10^–3^ – 1.0 × 10^–3^	-0.140	0.251	-8.8 × 10^–5^	-5.8 × 10^–5^ – 4.2 × 10^–5^	-0.039	0.749
	Men	-4.0 × 10^–3^	-8.0 × 10^–3^ – 1.0 × 10^–3^	-0.272	0.114	-4.9 × 10^–5^	-12.3 × 10^–5^ – 2.4 × 10^–5^	-0.232	0.181
	Women	-2.0 × 10^–3^	-4.0 × 10^–3^ – 4.0 × 10^–3^	-0.019	0.913	3.8 × 10^–5^	-2.6 × 10^–5^ – 10.1 × 10^–5^	0.210	0.233
IP	All	**-3.7 × 10**^**–2**^	**-5.2 × 10**^**–2**^** – -2.2 × 10**^**–2**^	**-0.513**	** < 0.001**	**-5.6 × 10**^**–4**^	**-7.6 × 10**^**–4**^** – -3.6 × 10**^**–4**^	**-0.571**	** < 0.001**
	Men	**-4.2 × 10**^**–2**^	**-8.5 × 10**^**–2**^** – -2.6 × 10**^**–2**^	**-0.679**	** < 0.001**	**-6.9 × 10**^**–4**^	**-9.3 × 10**^**–4**^** – -4.4 × 10**^**–4**^	**-0.707**	** < 0.001**
	Women	**-3.0 × 10**^**–2**^	**-4.2 × 10**^**–2**^** – -1.8 × 10**^**–2**^	**-0.664**	** < 0.001**	**-4.0 × 10**^**–4**^	**-6.6 × 10**^**–4**^** – -1.7 × 10**^**–4**^	**-0.524**	**0.001**
RF	All	**-6.0 × 10**^**–2**^	**-8.2 × 10**^**–2**^** – -3.7 × 10**^**–2**^	**-0.544**	** < 0.001**	**-8.8 × 10**^**–4**^	**-11.8 × 10**^**–4**^** – -5.9 × 10**^**–4**^	**-0.586**	** < 0.001**
	Men	**-6.7 × 10**^**–2**^	**-9.2 × 10**^**–2**^** – -4.3 × 10**^**–2**^	**-0.692**	** < 0.001**	**-10.7 × 10**^**–4**^	**-14.7 × 10**^**–4**^** – -6.8 × 10**^**–4**^	**-0.699**	** < 0.001**
	Women	**-5.0 × 10**^**–2**^	**-7.4 × 10**^**–2**^** – -2.7 × 10**^**–2**^	**-0.609**	** < 0.001**	**-6.7 × 10**^**–4**^	**-10.7 × 10**^**–4**^** – -2.7 × 10**^**–4**^	**-0.518**	**0.002**
QUAD	All	**-2.4 × 10**^**–1**^	**-3.6 × 10**^**–1**^** – -1.2 × 10**^**–1**^	**-0.432**	** < 0.001**	**-3.0 × 10**^**–3**^	**-4.5 × 10**^**–3**^** – -1.8 × 10**^**–3**^	**-0.491**	** < 0.001**
	Men	**-2.0 × 10**^**–1**^	**-2.9 × 10**^**–1**^** – -1.1 × 10**^**–1**^	**-0.609**	** < 0.001**	**-4.0 × 10**^**–3**^	**-5.6 × 10**^**–3**^** – -2.4 × 10**^**–3**^	**-0.669**	** < 0.001**
	Women	**-2.6 × 10**^**–1**^	**-3.7 × 10**^**–1**^** – -1.5 × 10**^**–1**^	**-0.643**	** < 0.001**	**-2.1 × 10**^**–3**^	**-3.6 × 10**^**–3**^** – -0.6 × 10**^**–3**^	**-0.442**	**0.009**

Bold font indicates significance

*IC* Iliocapsularis, *IP* Iliopsoas, *RF* Rectus femoris, *QUAD* quadriceps, *B* coefficient, *CI* confidence interval, *β* Standardized coefficient, *p* p-value

The results of the analysis for normalized CSA of IC were similar to those of CSA (sex: p = 0.03, partial *η*^2^ = 0.07; age: *p* = 0.96, partial η^2^ = 0.00), and normalized CSA of the IC in men groups was larger than that in women groups; however, there was no difference between the younger and older groups (Table 3). In addition, a larger normalized CSA of the other three muscles was found in the younger and in the men’s groups than that in the older and in the women’s groups (Table 3). The CSA of the IC, the IP, the RF, and the QUAD were significantly associated with body weight (standardized coefficients: 0.559, 0.666, 0.626, and 0.754, respectively; *p* < 0.001). The body weight is larger in men’s groups or younger groups than that in women’s groups or older groups (*p* < 0.001 and effect size (*r*) = -0.69 for men’s and women’s groups; *p* = 0.047 and effect size (*r*) = -0.24 for younger and older groups). Furthermore, linear regression analyses showed similar results to CSA. Normalized CSA of the IP, RF, and QUAD was significantly associated with age, but that of the IC was not associated with age. The regression analyses in only men, only women, and all individuals showed similar results (Table 4).

## Discussion

This study examined the number of individuals whose IC could be identified on MRI, and the effect of age and sex on the identification rate. In addition, the present study tested whether age and sex could be associated with the muscle CSA in the lower limb, including IC. As a result, IC was identified in 85–95% of individuals on MRI, and its percentage was not associated with age and sex. In addition, sex-related differences were found in the CSA of the IC, while no age-related difference was found in CSA of the IC. Furthermore, age and sex were associated with the CSAs of the IP, RF, and QUAD, with larger areas in men and younger groups than in women and older groups. These results did not change, even after normalizing the CSA by body weight. Additionally, regression analyses have shown significant associations between CSAs of other muscles and age, but not IC. The aforementioned results support our hypothesis. To the best of our knowledge, this study is the first to reveal age- and sex-related differences in IC size.

In general, muscle size is affected by age and sex [21], which is supported by our results for the IP, RF, and QUAD. On the other hand, this study shows that the CSA of the IC was not different between healthy older and younger individuals, although the CSA of the IC was significantly larger in men than in women. A previous study reported that the thickness and width of the IC were larger in men than in women, and thus supported the sex-related difference in the CSA of the IC in our study [13]. Ikezoe et al. reported that soleus muscle thickness was not affected by age, while the size of other leg muscles, including the psoas, RF, vasti, and triceps surae, decreased with age [14]. Ota et al. showed no age-related decrease in size in the transversus abdominis muscle among the abdominal muscles [15]. These authors mentioned that the muscles that are less susceptible to age-related changes have a high percentage of type I fibers and contribute to joint stability. The low susceptibility to age-related differences was shown in a high insulin-sensitive muscle fiber [24], and type I muscle fibers were highly insulin-sensitive [25]. The muscle size of the stabilizer may be maintained by low muscle activity during daily activities [15]. To our knowledge, there was no report on muscle fiber types in the IC. According to previous studies, the percentage of type I fibers was 49.2%, 29–43%, and 29–62% in the IP, RF, and QUAD, respectively, and was higher in the deep than the superficial layer within a muscle [26]. Given that the IC can be regarded as a deep fiber of the IP [12], the IC could have the highest percentage of type I fibers among the four measured muscles. In addition, the IC contributes to hip stability since the origin of the IC is the capsular of anterior hip, and the size of the IC is larger in hip dysplasia than in healthy adults [6, 8, 10]. Therefore, the CSA of the IC was unlikely to decrease with age, at least during healthy aging.

Although the results were similar for the normalized CSA and CSA, the effect size seemed to have decreased in the normalized CSA compared to that in CSA. Because a larger body size would affect the CSA, and because body weight is heavier in men or younger individuals than that in women or older individuals, we presumed that normalization for body weight would eliminate this body size effect on CSA. In particular, the sex-related difference in the CSA would be reduced by normalization for body weight because the difference in body weight is larger between sexes than that between age groups. Our results support previous studies that reported more obvious sex-related differences in the CSA than those in CSA normalized for body weight [27, 28].

This study confirms that the IC was not identified in 8 of the 77 individuals at the measured points on MRI. An anatomical study and studies using MRA reported identification of the IC in all individuals [6, 8, 10]. However, some studies have pointed out that the border between the IC and the IP is unclear, and that these two muscles are often depicted as one muscle group on MRI [11–13]. Therefore, no consensus exists regarding the percentage of IC that can be identified on MRI. The discrepancy between the results of the study using MRA and those of the present study using MRI would be due to the individual’s body weight. Heavier individuals have more connective tissue thickness, such as the fascia forming a border between muscles [29]. On average, the individuals in this study had a lower body weight (56.0 ± 8.7 kg) than the previous study measuring the size of the IC (control group: 26 individuals [men, 13]; body weight, 75 ± 20 kg) [10]. It is possible that the thickness of the fascia could be reduced in this study compared to the aforementioned study. Additionally, the thickness of the fascia was 0.5 mm [30] and 0.3–1.0. mm at the groin and anterior thigh region [30–32]. Accordingly, the connective tissue, such as the fascia, might be too thin for the MRI voxel size (0.5 × 0.5 × 4.0 mm) to clearly detect the border between the IC and IP in individuals whose IC could not be identified in the present study. Indeed, the IC was observed around the insertion and/or origin of the IC even in those individuals whose IC was not identified at the measured area (the center of the femoral head). Therefore, it is assumed that the IC was not absent in those individuals.

This study could acquire more detailed MRI images than the previous study since the slice thickness was the same as 4.00 mm; however, the magnetic field strength was stronger (3.0 T) than that in the previous study (1.5 T) [6, 10]. Even though detailed MRI was used, the identification of the IC was difficult in a few individuals. This suggests that the unidentifiable IC could be a limitation of MRI. While MRI is a useful way to evaluate the size of the IC as well as the diagnosis of hip pathologies, additional evaluation by a high-resolution ultrasound machine may allow us to measure the IC in all individuals [11, 33].

This study has a few limitations. First, it is possible that individuals with minor hip pathologies were included in this study. Although individuals with obvious bony deformities on MRI were excluded, individuals with diseases that were difficult to identify on MRI, such as acetabular labral lesions, may have been included. In these individuals, the pathology could affect the CSA of the IC. Second, the muscle size was calculated from one slice of MRI. The result might differ from a study in which the volume or maximal CSA is used in each muscle. In addition, we did not confirm the repeatability of the measurements. However, our method is reasonable since differences in IC size were detected between patients with acetabular dysplasia and healthy individuals in a different study that used the same procedure as in the current study [10]. Future research should focus on identifying the factors associated with the IC size, such as bone and joint morphology, and on determining age-related changes in other properties such as fat infiltration and muscle stiffness in the IC.

## Conclusions

In conclusion, the IC muscle can be discriminated in 85—95% of healthy individuals using MRI, and this percentage was not associated with sex or age. Although sex and age are associated with the CSA of lower limb muscles other than the IC, only sex is associated with the CSA of the IC. This result did not change even when body weight normalized the CSA.

## Data Availability

The datasets generated and/or analyzed during the current study are not publicly available due to ethical restrictions, but are available from the corresponding author on reasonable request.

## Abbreviations

ANOVA: Analysis of Variance
CSA: Cross-Sectional Area
IC: Iliocapsularis Muscle
ICC: Intraclass Correlation Coefficients
IP: Iliopsoas Muscle
MRA: Magnetic Resonance Arthrography
MRI: Magnetic Resonance Imaging
QUAD: Quadriceps
RF: Rectus Femoris
