# Correction: Age- and sex-related differences of muscle cross-sectional area in iliocapsularis: a cross-sectional study

**DOI:** 10.1186/s12877-022-03281-3

**Published:** 2022-08-10

**Authors:** Masahide Yagi, Masashi Taniguchi, Hiroshige Tateuchi, Tetsuya Hirono, Yoshihiro Fukumoto, Momoko Yamagata, Ryusuke Nakai, Yosuke Yamada, Misaka Kimura, Noriaki Ichihashi

**Affiliations:** 1 Human Health Sciences, Graduate School of Medicine, Kyoto University, 53 Kawahara-cho, Shogoin, Sakyo-ku, Kyoto, 606-8507 Japan; 2 Research Fellow of Japan Society for the Promotion of Science, Kojimachi Business Center Building, 5-3-1 Kojimachi, Chiyoda-ku, Tokyo, 102-0083 Japan; 3 School of Health and Sport Science, Chukyo University, 101 Tokodachi, Kaizu-cho, Toyota, Aichi 470-0393 Japan; 4 Faculty of Rehabilitation, Kansai Medical University, 18-89 Uyama Higashimachi, Hirakata, Osaka, 573-1136 Japan; 5 Institute for the Future of Human Society, Kyoto University, 46 Shimoadachi-cho, Yoshida Sakyo-ku, Kyoto, 606-8501 Japan; 6 National Institutes of Biomedical Innovation, Health and Nutrition, 1-23-1, Toyama, Shinjuku-ku, Tokyo, 162-8636 Japan; 7 Institute for Active Health, Kyoto University of Advanced Science, Nanjo Otani, 1-1 Sogabecho, Kameoka, Kyoto, 621-8555 Japan; 8 Faculty of Nursing, Doshisha Women’s College of Liberal Arts, Koudo, Kyotanabe, Kyoto, 610-0395 Japan

**Correction: BMC Geriatr 22, 435 (2022)**

**https://doi.org/10.1186/s12877-022-03127-y**

After publication of this article [1], the authors reported that the legend to Table 1 was incorrectly given as 'Overview of the study events for each participant during the follow-up period', but should have been 'Demographic data for each group' and the legend of Table 3 was incorrectly given as ‘Doses escalation during the study follow up’, but should have been ‘Results of two-way ANOVA’.

The original article [1] has been corrected.
